# The role of haemoglobin level in predicting the response to first-line chemotherapy in advanced colorectal cancer patients

**DOI:** 10.1038/sj.bjc.6603204

**Published:** 2006-06-13

**Authors:** M Tampellini, A Saini, I Alabiso, R Bitossi, M P Brizzi, C M Sculli, A Berruti, G Gorzegno, A Magnino, E Sperti, S Miraglia, L Forti, O Alabiso, M Aglietta, A Harris, L Dogliotti

**Affiliations:** 1 Department of Medical Oncology, University of Torino, San Luigi Hospital, 10043 Orbassano, Italy; 2 Department of Medical Oncology, University of Torino, IRCC Candiolo, Italy; 3 Department of Medical Oncology, University of Novara, Novara, Italy; 4 Department of Medical Oncology, University of Oxford, Oxford, UK

**Keywords:** anaemia, colorectal neoplasms, 5-fluorouracil, activity

## Abstract

The purpose of the study was to evaluate the influence of baseline haemoglobin level in predicting response to 5-fluorouracil (5FU)-based first-line chemotherapy in advanced colorectal cancer patients. Data from 631 patients were collected from three different institutions. Globally, overall response rate was 35.8% (226 out of 631). Factors influencing response rate were 5FU dose intensity (high: 43.1%, low: 34.0%, *P*=0.03); oxaliplatin (yes: 45.8%, no: 22.9%, *P*<0.0001), performance status (PS 0: 46.1%, 1: 28.8%, 2: 26.7%, *P*<0.0001), and haemoglobin levels (⩾12 g dl^−1^: 40.4%, <12 g dl^−1^: 29.2%, *P*=0.004). In subgroup analysis significant differences in response rate between anaemic and nonanaemic patients were recorded in those patients treated with infusional chemotherapies (45.7 *vs* 25.5%, *P*<0.0001), with high 5FU dose intensity (50.3 *vs* 32.7%, *P*=0.005), with PS=0 (49.8 *vs* 37.9%, *P*=0.03), and with liver metastases (44.8 *vs* 33.8%, *P*=0.002), whereas no difference was evident in those subjects treated with bolus schedules or according to gender. Anaemia was a strong predictor for activity of first-line 5FU-based chemotherapy especially in those groups that showed the best responses, for example high performance status, infusionally treated, higher 5FU dose and those with liver secondaries. Patients with higher haemoglobin levels recorded a greater response rate and a longer time to progression and survival than anaemic subjects. Prospective evaluation of role of correcting anaemia on response to therapy is justified by these results.

Malignant neoplasms of the large bowel are a leading health problem in the western countries, representing the second most frequent cause of cancer-related death (Parkin, 2001). The backbone of treatment for colorectal cancer is 5-fluorouracil (5FU), a fluorinated pyrimidine (Meyerhardt and Mayer, 2005). The biochemical pharmacology of 5FU is well understood and has been extensively reviewed (Pinedo and Peters, 1988; Diasio and Harris, 1989). 5FU is a prodrug which requires anabolism for its cytotoxic effects. Several anabolic pathways are recognized: primarily conversion to FdUMP, which inhibits thymidylate synthase (TS), the rate-limiting enzyme in pyrimidine nucleotide synthesis; conversion to FUTP which can be incorporated to RNA; conversion to FdUTP which can be incorporated into DNA. Enzymes involved into these different pathways include thymidine phosphorylase (TP) and thymidine kinase (TK). Furthermore, 5FU is able to induce apoptosis in normal and tumoural intestinal cells (Pritchard *et al*, 1988; Nita *et al*, 1998).

The overall response rate and the survival of metastatic colorectal patients have been dramatically increased in the last decade with the introduction of new active drugs, such as oxaliplatin, irinotecan and more recently cetuximab and bevacizumab, in combination with 5FU and capecitabine (Meyerhardt and Mayer, 2005). Response rate increased from 15 to 25% of the 1990s up to 50–60% and overall survival passed from 12 months to more than 20 months (Levi *et al*, 1999; Tournigand *et al*, 2004). Interestingly, several meta-analyses analysing pooled data coming from phase III trials on first-line treatment demonstrated that an increase in tumour control translates into a gain in overall survival (Buyse *et al*, 2000; Louvet *et al*, 2001). This finding seems to justify further evaluation of new treatment strategies focused to increase tumour response rate.

Anaemia is common in patients with cancer. Its prevalence was reported to be 70–85% in patients with Hodgkin disease and 40–80% in patients with advanced colorectal cancer, according to the cutoff considered (Knight *et al*, 2004). Haemoglobin level has been reported to impact on chemotherapy outcomes in a number of malignancies, particularly in breast (Bottini *et al*, 2003) and other gynaecological cancers or in head and neck cancer, especially when associated with radiotherapy (Koukourakis *et al*, 2000; Chua *et al*, 2004; Winter *et al*, 2004). Most studies have reported low pretreatment Hb levels to be a poor prognostic factor for tumour control and/or patients survival. These findings are consistent with experimental data. Several *in vitro* studies, in fact, demonstrated that cancer cells that are sensitive to 5FU when normally oxygenated may become resistant in hypoxic conditions (Teicher *et al*, 1981; Tannock, 1987; Grau and Overgaard, 1992).

The mechanism for this resistance has not been clarified yet. It has been demonstrated that low haemoglobin levels resulted in peripheral tissue hypoxia (Vaupel and Harrison, 2004), that may produce cell death if severe or prolonged. Although hypoxia is toxic to both cancer cells and normal cells, cancer cells undergo genetic and adaptive changes that allow them to survive and even proliferate in a hypoxic environment (Harris, 2002). Hypoxia regulates different pathways including angiogenesis, growth factor signalling, immortalisation, tissue invasion and metastasis, and apoptosis. In particular, vascular endothelial growth factor (VEGF)-A is a key factor (Harris, 2002). Vascular endothelial growth factor is thought to be the most potent and specific stimulator of angiogenesis and has been demonstrated to be associated with recurrence, metastasis and prognosis in colorectal cancer patients (Takahashi *et al*, 1995; Guba *et al*, 2004). Moreover, hypoxia may attract macrophages, which deliver TNF*α* and interleukin 1 in the tumour microenvironment. These inflammatory cytokines upregulate the expression of TP (TP – also called platelet-derived endothelial cell growth factor (PD-ECGF)), one of the key enzymes for fluoropyrimidine metabolism, which acts also as an antiapoptotic factor (Toi *et al*, 2005).

This body of clinical and preclinical evidences prompted us to investigate whether haemoglobin levels at baseline can predict response to first-line 5FU-based chemotherapy in patients with advanced colorectal cancer.

## MATERIALS AND METHODS

### Patients

Patients were identified from the central databases of three different institutions in which these patients were treated and followed from June 1993 to April 2004. These institutions were: (1) Università degli Studi di Torino, Oncologia Medica, ASO San Luigi di Orbassano; (2) Università degli Studi di Torino, IRCC Candiolo; (3) Università degli Studi ‘G. Avogadro’, ASO Maggiore della Carità, Novara. Patients who met the following criteria were selected for this study: (1) first diagnosis of metastatic colorectal cancer; (2) first-line 5FU-based chemotherapy; (3) haemoglobin determination within 1 week before first-line chemotherapy; (4) at least one clinical response determination during therapy.

Response evaluation was performed according to each single institution experience. It included at least clinical examination, complete abdominal computed tomography or ultrasound scan, and thoracic computed tomography scan or standard radiography. Other radiological exams or biochemical determinations were performed according to symptoms and metastatic sites.

Treatment response was classified according to the UICC criteria (Miller *et al*, 1981). A complete response was defined as the complete disappearance of all clinically detectable malignant disease. A partial response was characterized as a decrease ⩾50% in the sum of the products of the two longest perpendicular diameters of all measurable lesions. Progressive disease was defined as an increase of at least 25% in the size of measurable lesions and the development of new lesions. Only the best tumour response was recorded.

Time to progression and overall survival were estimated from treatment start till progression, death or date of the last follow-up (30 April 2004). Patients not progressing, alive or lost to follow-up at the time of data computation were censored at the time of the last follow-up examination.

### Data collection

In each institution, a team of three physicians filled in a standardized electronic sheet including major demographic and prognostic characteristics for every consecutive patient who met the inclusion criteria. Data were abstracted from patient charts and clinical records.

In order to assure good quality of the data, clinical response to therapy was re-evaluated by three physicians (one from each institution). As a whole, the radiological images of 290 patients (46%) randomly chosen from the entire data set were reviewed, with a concordance between recorded and re-evaluated responses of 97%.

Electronic sheets were verified at the end of data collection to eliminate data entry errors and were subsequently entered into a computerized database, which represented the source file for this study.

### Statistical analyses

The area under the receiver operating characteristic curve (ROC) was calculated as a measure of predictive discrimination of tumour response by haemoglobin levels. An index of 0.5 indicates no discrimination ability, whereas a value of 1 indicates perfect discrimination.

Differences between groups of parametric variables were validated by the *t* test for means. The difference between proportions was evaluated by the *χ*^2^ test with Yates' correction, when necessary. Survival curves were plotted using the Kaplan–Meier method and were statistically evaluated using the log-rank test. Multivariate survival analysis according to the Cox model was performed to eliminate confounding variables. Martingale and Schoenfeld residuals were used to check the adequacy of the linearity and the proportional hazard assumptions (Harrell *et al*, 1984).

These statistical computations were performed using the SPSS for Windows and STATISTICA for Windows software.

## RESULTS

### Patient characteristics

A total of 631 patients met the inclusion criteria and were considered in the study. Their characteristics are outlined on Table 1. Median age was 61.2 years, 385 were male (61%) and most of them were in good performance status (PS 0–1) at the time of their first recurrence. More than a half of this study group presented with synchronous metastases and 172 patients (27.3%) received one line of adjuvant therapy. No patient received oxaliplatin or irinotecan in the adjuvant setting. At study entry, mean haemoglobin value was 12.5 g dl^−1^ (standard error of the mean±0.07 g dl^−1^). The relationship between haemoglobin levels and various parameters are shown on Table 1. Differences were evident when patients were stratified according to gender, performance status, and whether or not they presented with liver or lung metastases.

### Chemotherapy regimens

Table 2 summarizes the types of chemotherapy administered to the study population. Chronomodulated chemotherapy was administered to 296 patients, whereas 205 of them received one of the FOLFOX or FOLFIRI regimens (Tournigand *et al*, 2004). Infusional 5FU was administered to 380 patients, a combination of bolus+infusional 5FU to 211 patients and bolus 5FU to 40 patients. In the subsequent analyses, these two latter groups were considered together to improve statistical power. Finally, 356 patients received 5FU combined with oxaliplatin and 50 with irinotecan. The 5FU dose intensity was calculated for each patients dividing the amount of the drug actually administered, expressed as milligrams per square meter, by the time between chemotherapy onset and the date of the last dispensed cycle, expressed as the number of weeks between the two time points. Median 5FU dose intensity was 1165 mg sqm week^−1^ (range 96–4248). Differences in median 5FU dose intensities were found when patients were stratified according to the type of infusion (bolus group: 967 mg sqm week^−1^; infusional group: 1341 mg sqm week^−1^, *P*=0.02) and whether or not they received oxaliplatin (yes: 1225 mg sqm week^−1^; no: 1037 mg sqm week^−1^, *P*=0.01). A lower median 5FU dose intensity was administered to patients with PS 1 (1070 mg sqm week^−1^) than those with PS 0 (1217 mg sqm week^−1^) or PS 2 (1212 mg sqm week^−1^). This last difference, however, was not statistically significant.

### Response to chemotherapy

After chemotherapy, 226 patients obtained a tumour shrinkage >50% (overall response rate: 35.8%), 259 (41.1%) did not present significant tumour changes within 3 months, and 146 progressed (23.1%).

Univariate analyses of response rate and time to progression according to various parameters are summarized on Table 3.

The World Health Organization (WHO) recognized as anaemic, women with haemoglobin levels below 12.0 g dl^−1^ and men with levels below 13.0 g dl^−1^ (WHO/UNICEF/UNU, 2001). Considering these indications, the mean haemoglobin value of the study population (12.5 g dl^−1^), and the response rate of patients stratified by haemoglobin levels, the haemoglobin cutoff to discriminate anaemic *vs* nonanaemic patients in this study was determined to be 12.0 g dl^−1^.

Patients receiving a 5FU dose intensity higher than 1165 mg sqm week^−1^ had a higher response rate (43.1 *vs* 34.0%; *X*^2^=4.5, *P*=0.03) and a longer time to progression (TTP) (11.6 *vs* 10.5 months, *P*=0.07), even if this latter analysis did not reach a fully statistical validation. Similar results were recorded in patients receiving oxaliplatin (45.8 *vs* 22.9%, *X*^2^=35.3, *P*<0.0001; TTP 11.6 *vs* 9.8 months, *P*=0.004), whereas they were not evident for those treated with irinotecan (28.0 *vs* 36.5%, *X*^2^=1.1, *P*=NS). Patients with the best performance status responded better than those in worse conditions. Response rates, in fact, were 46.1, 28.8, and 26.7%, respectively (*P*<0.0001). No difference in response rate was found between patients treated with infusional 5FU regimens (141 out of 380, 37,1%) and those receiving bolus 5FU (85 out of 251, 33.9%; *X*^2^ *P*=0.4), whereas the corresponding times to progression were 11.8 *vs* 10.0 months (*P*=0.004). Finally, male patients presented the same chance to respond to therapy as female subjects (36.1 *vs* 35.4%; *X*^2^ *P*=0.8).

### Treatment response related to haemoglobin levels and drug regimen

**Figure 1:**
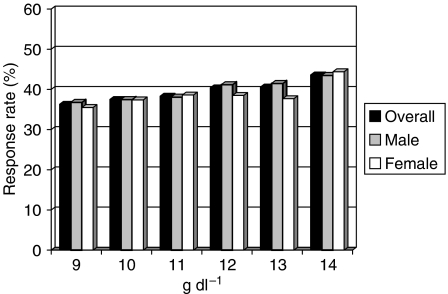
Response rate by 1 g dl^−1^ increments of haemoglobin levels overall and stratified by gender in the entire study population.

Figure 1 shows response rate by subgroup analyses according to 1 g dl^−1^ increments of haemoglobin levels, gender and type of infusion. Overall, at each increment a higher response rate was recorded, 43.6% being the highest at 14 g dl^−1^ cutoff. However, a plateau between 12.0 and 14.0 g dl^−1^ was evident with response rate ranging from 40.4 to 43.6%. No difference between genders was recorded.

When analysing patients treated with bolus regimens, response rates were similar regardless of gender and haemoglobin levels. A different pattern, however, was evident in those patients treated with infusional schedule. In fact, at each haemoglobin increment corresponded a higher response rate. This was particularly true for male patients, in which the threshold of 14 g dl^−1^ was able to discriminate the highest proportion of responding subjects, whereas in female patients a plateau was evident at 12 g dl^−1^.

Overall response rates according to the <12 g cutoff were 29.2% (75 out of 257) in anaemic patients and 40.4% (151 out of 374) in nonanaemic ones (*X*^2^=8.30; *P*=0.004), whereas the times to progression were 10.0 *vs* 11.7 months (*P*<0.001), and the median overall survivals were 26.4 *vs* 20.2 (*P*<0.0001). When considering all the patients obtaining a clinical response or a disease stabilization after chemotherapy, the same figures were 67.3% (173 out of 257) and 83.4% (312 out of 374), respectively (*X*^2^=22.22; *P*<0.0001).

The area under the ROC curve calculated in the entire study population to test the ability of haemoglobin values to discriminate the patients destined to obtain a clinical response was 0.57 (95%CI 0.53–0.62, *P*=0.001). Subgroup analyses revealed that the largest area was found when computing the ROC curve in those patients receiving infusional 5FU (0.64, 95%CI 0.58–0.69, *P*<0.001), whereas this test did not describe a discrimination ability in those patients treated with bolus 5FU. Taking into account these curves, the best sensitivity/specificity ratio was found to be for haemoglobin levels ranging between 11.0 and 13.0 g dl^−1^.

### Progression and survival related to anaemia classified as <12.0 g dl^−1^

Table 4 summarizes univariate response rate, time to progression, and overall survival analyses for patients stratified according to various parameters and haemoglobin levels. When patients were stratified according to the modality of 5FU administration, response rates were 25.5% (41 out of 161) in anaemic and 45.7% (100 out of 219) in nonanaemic patients receiving an infusional chemotherapy (*X*^2^=16.22; *P*<0.0001), whereas the same figures were 35.4% (34 out of 96) and 32.9% (51 out of 155) in those receiving bolus plus infusional 5FU (*P*=NS), respectively. Patients with haemoglobin values ⩾12.0 g dl^−1^ presented a longer time to progression either in the infusional subgroup (13.0 *vs* 10.4 mo, *P*=0.007) and in the bolus subgroup (10.1 *vs* 9.1 months, *P*=0.02), respectively, whereas a difference in overall survival was evident only in those patients submitted to an infusional regimen (27.5 *vs* 20.0 months, *P*<0.0001). When considering all the patients obtaining a clinical response or a disease stabilization after chemotherapy, the response rates were: infusional subgroup: anaemic 68.3% (110 out of 161) *vs* nonanaemic 86.3% (189 out of 219) (*X*^2^=17.88, *P*<0.0001); bolus subgroup: anaemic 65.6% (63 out of 96) *vs* nonanaemic 79.4% (123 out of 155) (*X*^2^=5.82, *P*=0.015).

Among patients receiving more than 1165 mg sqm week^−1^ of 5FU, those with normal haemoglobin levels had a higher response to chemotherapy (50.3 *vs* 32.7%, *P*=0.005). This difference was not found for patients who received a lower 5FU dose intensity (34.9 *vs* 32.7%, respectively. *P*=NS). Similar results were found when considering the time to progression. Patients with normal haemoglobin levels survived longer regardless the administered dose of 5FU.

Differences in response rates were also found between anaemic and nonanaemic patients when they were stratified according whether or not they had received a chemotherapy containing oxaliplatin. In fact, in those patients receiving oxaliplatin the response rates were 39.4 *vs* 50.0% (*P*=0.05), whereas the corresponding figures in those patients not receiving oxaliplatin were 18.1 *vs* 27.5% (*P*=0.03), respectively.

When patients were stratified according to their performance status, a clear difference between anaemic and nonanaemic patients was evident in the subgroup with PS 0 (49.8 *vs* 37.9%, *P*=0.03), whereas there was a trend in that with PS 2 (44.0 *vs* 19.0%, *P*=0.16) and no difference in those patients with PS 1 (27.7 *vs* 27.4%).

### Multivariate analysis of haemoglobin as a continuous variable related to response and survival

Multivariate logistic regression analysis confirmed haemoglobin levels at baseline (as a continuous variable), oxaliplatin, liver metastasis and PS as independent predictive factors for response to first-line chemotherapy. Gender, age, stage at diagnosis, tumour grading, disease free interval, 5FU dose intensity, metastasis in lung or abdomen and number of involved sites did not enter the model (Table 5a).

Similar results were evident when performing the multivariate survival analysis according to the Cox model, with the only expected exception of the presence of lung metastasis as independent positive factor for prolonged survival (Table 5b).

## DISCUSSION

Anaemia was a strong predictor for activity of first-line 5FU-based chemotherapy in 631 advanced colorectal cancer patients. Both the ROC analysis and the multivariate model demonstrated that patients with higher haemoglobin levels had a greater chance of response to therapy. Although this study is retrospective and involves several institutions, the observations provide a necessary basis to consider prospective studies to evaluate the role of transfusion or erythropoietin in this context.

The activity of first-line chemotherapy in our study population is similar to that reported in the literature (Meyerhardt and Mayer, 2005). Response rates were 22.9% in those patients receiving 5FU with or without folinic acid, and 45.8% in those receiving also oxaliplatin. In our study, a high proportion of patients (56.4%) had an oxaliplatin-containing regimen, whereas few patients received irinotecan (7.9%). This was because institution #1 participated in several clinical phase II–III studies aiming to validate oxaliplatin-based chronochemotherapy in the early 1990s (Levi *et al*, 1994, 1997), and that institution #3 started to administer oxaliplatin before it was registered in the Italian market. This hampered any conclusion on the possible effect of anaemia on the activity of irinotecan.

It is well known that haemoglobin distributions vary with age, gender and with altitude and smoking. This observation was confirmed in our patients (Table 1). The WHO defined haemoglobin levels below which anaemia is present in an adult population as 12.0 g dl^−1^ in women and 13 g dl^−1^ in men (WHO/UNICEF/UNU, 2001). In the literature, many authors reported as ‘mild’, anaemic cancer patients with haemoglobin levels below 11 g dl^−1^, while those with levels below 9 g dl^−1^ were defined as with ‘severe’ anaemia (Knight *et al*, 2004). According to this evidence and to the computed ROC curve of our series that indicated the best cutoff of haemoglobin being between 11.0 and 13.0 g dl^−1^, we decided to classify as anaemic all the patients with values below 12.0 g dl^−1^. According to the response rates recorded dividing patients by 1 g dl^−1^ increment (Figure 1), while this cutoff might be ideal for women, it may be questionable for male patients, in which the highest chance to respond to therapy was recorded in those with haemoglobin levels above 14 g dl^−1^. For treatment purposes (e.g. blood transfusions or erythropoietin therapy), however, 12 g dl^−1^ is a reasonable target, 14 g dl^−1^ being at risk of higher side effect incidence. Finally, no difference according to gender was evident in the multivariate analyses of response and survival (Table 5) demonstrating that gender did not affect response rate.

With this relatively high cutoff we tried to reduce the impact of potential biases of this study linked to the observation, confirmed in our series (Table 3), that subjects with low haemoglobin levels have low quality of life scores when compared to subjects with greater levels (Holzner *et al*, 2002), and this condition might not permit aggressive oncological approaches. Interestingly, when considering only patients with PS 0, anaemic patients had a lower response rate than those with normal haemoglobin levels, suggesting an independent impact of these two factors. Multivariate regression analysis, in fact, together with oxaliplatin administration and the presence of liver metastases, confirmed performance status and haemoglobin levels as independent variables in determining response (Table 5). It has to be underlined that in this analysis haemoglobin levels were considered as continuous variable. Thus, the results obtained suggested that the higher haemoglobin level, the higher chances to respond to chemotherapy patients presented.

How anaemia may impact on treatment outcome is not clear. One way that cells respond to reduced oxygen levels is through hypoxia-inducible factor-1 (HIF-1) (Harris, 2002). Hypoxia-inducible factor-1 is a key transcription factor that regulates many pathways including angiogenesis, growth-factor signalling, tissue invasion and metastasis. Hypoxia-inducible factor-1 accumulation induces the gene expression of VEGF, of VEGF receptor 1 and can also lead to reduced expression of antiangiogenic proteins such as thrombospondin-1 and –2 (Harris, 2002). Vascular endothelial growth factor acts as a powerful mitogen to endothelial cells, induces vascular permeability, increases microvessel density, increases the incidence of metastasis, and decreases the apoptotic index upregulating endothelial antiapoptotic proteins (Perrone *et al*, 2004). Violette *et al* (2002) demonstrated that the resistance of colon cancer cells to long-term 5FU exposure *in vitro* is correlated to the relative level of bcl-2 in addition to the status of other proteins involved into the apoptotic pathways.

A second mechanism probably involved in drug resistance is through the upregulation of the gene expression of PD-ECGF also known as TP (Harris, 2002) mediated by several cytokines such as tumour necrosis factor-*α*, interleukin 1, and interferon-*δ* typically associated with macrophage infiltration which is often related to hypoxia (Lewis and Murdoch, 2005; Toi *et al*, 2005). Thymidine phosphorylase has been repeatedly reported to be associated *in vivo* with nonresponse to 5FU (Metzger *et al*, 1998; Ackland and Peters, 1999; Salonga *et al*, 2000; van Triest *et al*, 2000). This could be explained in several ways: similarly to VEGF and other growth factor induced by hypoxia, TP might act as an angiogenesis factor with reduction of the apoptotic propensity in tumour cells (Lu and Tanigawa, 1997). Alternatively, TP might increase the incorporation of thymine into DNA independently from the activity of TS, the target enzyme of 5FU, representing a possible rescue pathway against 5FU cytotoxicity. This mechanism, however, has been repeatedly questioned and new *in vivo* studies focusing on this possible mechanism are required (Patterson *et al*, 1998; Ackland and Peters, 1999; de Bruin *et al*, 2003). Finally, higher TP levels might divert 5FU to DNA rather than RNA pathway, the latter being possibly linked to an oxygen-independent cell death mechanism. It has been shown, in fact, that the level of 5FU incorporated into RNA is significantly higher in patients treated with bolus intravenous injection (bolus group) than in those who received continuous drug infusion (continuous group), whereas the TS inhibition was similar in both groups. Thus, cytotoxicity of 5FU was explained by RNA and DNA damage in the bolus group and only by DNA damage in the continuous group (Kubota *et al*, 2002; Noordhuis *et al*, 2004). The reactions catalysed by TP (i.e. the formation of 5-fluoro-deoxyuridine from 5FU and of thymine from thymidine) are involved in the 5FU pathways that result in TS inhibition and consequently in the DNA damage, whereas they have little or no effects on the RNA formation. Moreover, the decrease in proliferation index induced by hypoxia, may explain the survival of cancer cells in which the DNA has been damaged by 5FU, whereas a defect in the expression of either mRNA or rRNA due to 5FU might represent a mechanism of cell death less modified by hypoxia. This is consistent with our data which interestingly showed that the most striking difference in response rate between anaemic and nonanaemic patients was evident only in those subjects treated with infusional chemotherapy.

In our study only 40 patients (6.3%) received a 5FU bolus regimen. This sample was too small for any statistical inference and these subjects were pooled together with those receiving a combination of bolus plus infusional 5FU. Considering this last subgroup, while no disparity was evident in response rate, a significative difference between anaemic and nonanaemic patients was found when we considered the fraction of those patients obtaining either clinical response or stabilisation of the disease. It would be of great interest to perform similar analyses in those patients submitted to a pure, active 5FU bolus schedule like the IFL or the AIO regimens to determine whether this difference in response is due to biochemical reasons or simply to the fact that the Mayo or the Machover schedules are therapies with low activity.

Finally, it should be noted the effect of anaemia was detected in the groups with optimal criteria for response, for example, high performance status and high drug intensity, but with less active regimens or iller patients no effect was seen. It is likely that with an already low response rate effects of further detrimental factors will be harder to detect and less influential if outcome is already poor. This emphasizes the importance of delivering optimal therapy to those most likely to benefit. In view of recent controversies on erythropoietin (Bohlius *et al*, 2005), it is not clear whether haemoglobin should be maintained by transfusion or erythropoietin therapy, our results suggest this is an important topic for a randomised trial.

In conclusion, our data suggest that it would be of great importance to maintain adequate haemoglobin levels to obtain the best activity of first-line chemotherapy regimens, especially those that foresee the infusional administration of 5FU.

## Figures and Tables

**Table 1:** Patients' characteristics

**Total number of patients**	**631**
Patients from centre #1	352
Patients from centre #2	53
Patients from centre #3	226
Male/female	385/246
Median age (range)	61.2 years (28.9–84.9)
Colon/rectum	464/167
PS 0/1/2/unknown	337/156/30/108
	
*Stage at diagnosis*	
A	2
B	72
C	169
D	385
Unknown	3
	
*Previous adjuvant therapy*	
Yes/no	172/459
	
*Metastatic site at study entry*	
1 mts site	450 (71.3%)
>1 mts site	181 (28.7%)
Liver	457/631 (72.4%)
Lung	165/631 (26.1%)
Abdomen	217/631 (34.4%)
Bone	7/631 (1.1%)
	
Mean Hb level (±s.e.m.)	12.5 (±0.07) g dl^−1^
	
Male	12.8 (±1.8)
Female	12.0 (±1.5) (*P*<0.0001)
	
Colon	12.5 (±0.08)
Rectum	12.6 (±0.14) (*P*=NS)
	
PS 0	12.8 (±0.09)
PS 1	12.2 (±0.14)
PS 2	11.3 (±0.33) (*P*<0.0001)
	
1 mts site	12.5 (±0.08)
>1 mts site	12.4 (±0.14) (*P*=NS)
	
Liver mts	12.4 (±0.08)
No liver mts	12.7 (±0.13) (*P*=0.03)
	
Lung mts	12.4 (±0.08)
No lung mts	12.9 (±0.13) (*P*<0.01)

mts=metastasis; NS=not significant.

**Table 2:** First-line chemotherapy

**Schedule**	**5FU administration**	**No. of patients**
Bolus FUFOL	Bolus	22
LV5FU2	Bolus+infusional	31
5FU continuous infusion	Infusional	49
FOLFOX2	Infusional	25
FOLFOX4	Bolus+infusional	61
FOLFOX6	Bolus+infusional	78
FOLFIRI	Bolus+infusional	41
Chronomodulated FF	Infusional	110
Chronomodulated FFL	Infusional	186
Various bolus	Bolus	18
Various infusional	Infusional	10

LV5FU2: folinic acid 200 mg sqm day^−1^ 2 h infusion; 5FU 400 mg sqm day^−1^ bolus; 5FU 600 mg sqm day^−1^ 22 h infusion d1-2q14d

FOLFOX2: oxaliplatin 100 mg sqm^−1^ d1; folinic acid 300 mg sqm day^−1^ in 2 h infusion; 5FU 1500–1800 mg sqm day^−1^ in 22 h infusion d1-2q14d

FOLFOX4: oxaliplatin 85 mg sqm^−1^ d1; folinic acid 200 mg sqm day^−1^ 2 h infusion; 5FU 400 mg sqm day^−1^ bolus; 5FU 600 mg sqm day^−1^ 22 h infusion d1-2q14d

FOLFOX6: oxaliplatin 100 mg sqm d1; folinic acid 200 mg sqm^−1^ 2 h infusion; 5FU 400 mg sqm^−1^ bolus; 5FU 2400–3000 mg sqm^−1^ 46 h infusion

FOLFIRI: irinotecan 180 mg sqm^−1^ d1; folinic acid 200 mg sqm day^−1^ 2 h infusion; 5FU 400 mg sqm day^−1^ bolus; 5FU 600 mg sqm day^−1^ 22 h infusion d1-2q14d

Chronomodulated FF: folinic acid 300 mg sqm day^−1^; 5FU 700–1000 mg sqm day^−1^ d1-4q14d

Chronomodulated FFL: oxaliplatin 25 mg sqm day^−1^; folinic acid 300 mg sqm day^−1^; 5FU 700–1000 mg sqm day^−1^ d1-4q14d.

**Table 3:** Univariate analyses of response to therapy, time to progression and overall survival

	**Response rate**	***P***	**TTP**	***P***	**OS**	***P***
Hb levels⩾12 g dl^−1^	40.4% (151/374)		11.7		26.4	
Hb levels<12 g dl^−1^	29.2% (75/257)	0.004	10.0	<0.001	20.2	<0.0001
						
5FU DI ⩾1165 mg sqm week^−1^	43.1% (110/255)		11.6		24.9	
5FU DI<1165 mg sqm week^−1^	34.0%(86/253)	<0.0001	10.5	0.07	25.2	NS
						
Therapy with oxaliplatin	45.8% (163/356)		11.6		25.1	
Therapy without oxaliplatin	22.9% (63/275)	<0.0001	9.8	0.004	22.3	<0.001
						
Therapy with irinotecan	28% (14/50)		9.5		25.3	
Therapy without irinotecan	36.5% (212/581)	NS	10.9	NS	23.3	NS
						
Infusional regimen	37.1% (141/380)		11.8		23.9	
Bolus+infusional regimen	33.5% (84/251)	NS	10.0	0.004	23.3	NS
						
PS 0	46.1% (154/334)		11.9		29.0	
PS 1	28.8% (45/156)		9.9		20.1	
PS 2	26.7% (8/30)	<0.001	7.6	<0.001	11.0	<0.0001
						
Liver mts	38.7% (177/457)		10.8		23.3	
No liver mts	28.2% (49/174)	0.01	10.9	NS	24.7	NS
						
Lung mts	40.0% (66/165)		13.1		30.0	
No lung mts	34.3% (160/466)	NS	10.4	<0.001	22.2	<0.001
						
Male	36.1% (139/385)		11.0		24.3	
Female	35.4% (87/246)	NS	10.6	NS	23.2	NS

mts=metastasis; NS=not significant; OS=overall survival (months); TTP=time to progression (months).

**Table 4:** Univariate analyses of response to therapy, time to progression, and overall survival according to haemoglobin levels

	**RR**	***P***	**TTP**	***P***	**OS**	***P***
*5FU DI ⩾1165 mg sqm week* ^−*1*^						
Hb levels ⩾12 g dl^−1^	50.3% (76/151)		12.8		28.1	
Hb levels<12 g dl^−1^	32.7% (34/104)	0.005	10.1	0.002	22.0	<0.01
						
*5FU DI<1165 mg sqm week* ^−*1*^						
Hb levels ⩾12 g dl^−1^	34.9% (52/149)		10.8		27.8	
Hb levels<12 g dl^−1^	32.7% (34/104)	0.7	10.2	0.33	21.6	<0.05
						
*Infusional regimen*						
Hb levels ⩾12 g dl^−1^	45.7% (100/219)		13.0		27.5	
Hb levels<12 g dl^−1^	25.5% (41/161)	<0.0001	10.4	0.007	20.0	<0.0001
*Bolus*+*infusional regimen*						
Hb levels ⩾12 g dl^−1^	32.9% (51/151)		10.1		24.3	
Hb levels<12 g dl^−1^	35.4% (34/96)	0.8	9.1	0.02	20.3	NS
						
*Therapy with oxaliplatin*						
Hb levels ⩾12 g dl^−1^	50.0% (107/214)		12.8		28.4	
Hb levels<12 g dl^−1^	39.4% (56/142)	0.05	10.5	0.002	22.1	<0.01
						
*Therapy without oxaliplatin*						
Hb levels ⩾12 g dl^−1^	27.5% (44/160)		10.6		24.6	
Hb levels<12 g dl^−1^	18.1% (19/105)	0.03	8.6	0.09	18.3	<0.001
						
*PS 0*						
Hb levels ⩾12 g dl^−1^	49.8% (110/221)		13.5		30.1	
Hb levels<12 g dl^−1^	37.9% (44/116)	0.03	10.8	0.003	25.3	<0.05
*PS 1*						
Hb levels ⩾12 g dl^−1^	27.7% (23/83)		9.6		22.3	
Hb levels<12 g dl^−1^	27.4% (20/73)	NS	10.0	NS	16.3	NS
*PS 2*						
Hb levels ⩾12 g dl^−1^	44.4% (4/9)		8.5		10.9	
Hb levels<12 g dl^−1^	19.0% (4/21)	0.16	7.0	NS	10.9	NS
						
*Liver mts*						
Hb levels ⩾12 g dl^−1^	44.8% (116/259)		12.0		25.9	
Hb levels<12 g dl^−1^	30.8% (61/198)	0.002	9.9	<0.001	20.1	<0.001
						
*No liver mts*						
Hb levels ⩾12 g dl^−1^	30.4% (35/115)		11.2		26.6	
Hb levels<12 g dl^−1^	23.7% (14/59)	NS	10.4	NS	19.0	<0.05
						
*Lung mts*						
Hb levels ⩾12 g dl^−1^	44.1% (49/111)		14.2		35.1	
Hb levels<12 g dl^−1^	31.5% (17/54)	0.12	11.6	0.04	25.1	<0.01
						
*No lung mts*						
Hb levels ⩾ g dl^−1^	38.8% (102/263)		11.2		23.7	
Hb levels<12 g dl^−1^	28.6% (58/203)	0.02	9.8	0.01	18.3	<0.01
						
*Male*						
Hb levels ⩾12 g dl^−1^	41.2% (106/257)		11.8		27.5	
Hb levels<12 g dl^−1^	25.8% (33/128)	0.03	9.4	<0.001	18.8	<0.0001
						
*Female*						
Hb levels ⩾12 g dl^−1^	38.5% (45/117)		11.5		24.6	
Hb levels<12 g dl^−1^	32.6% (42/129)	NS	10.2	0.05	21.6	0.05

mts=metastasis; NS=not significant.

**Table 5:** Multivariate analyses

	***β***	**Standard error of *β***	***P*-level**
*(a) Logistic multivariate regression analysis for clinical response*			
Liver metastasis	0.097	0.043	0.02
Haemoglobin value	0.096	0.044	0.03
Performance status	−0.130	0.044	0.003
Chemotherapy with oxaliplatin	0.144	0.043	0.0001
			
*(b) Multivariate survival analysis according to the Cox model*			
Lung metastasis	−0.322	0.118	0.006
Haemoglobin value	−0.127	0.029	<0.0001
Performance status	0.436	0.088	<0.0001
Chemotherapy with oxaliplatin	−0.256	0.100	0.01

Lung metastasis: 1 yes; 0 no; Liver metastasis: 1 yes; 0 no

Haemoglobin values as a continuous variable.

Performance status: 0, 1, 2.

Chemotherapy with oxaliplatin: 1 yes; 0 no.

Gender, age, stage at diagnosis, tumour grading, disease-free interval, 5FU dose intensity, metastasis in liver or abdomen and number of involved sites did not enter the model.
